# Canopy and Understory Nitrogen Addition Alters Organic Soil Bacterial Communities but Not Fungal Communities in a Temperate Forest

**DOI:** 10.3389/fmicb.2022.888121

**Published:** 2022-06-10

**Authors:** Yang Liu, Xiangping Tan, Shenglei Fu, Weijun Shen

**Affiliations:** ^1^Ecological Conservation and Restoration Laboratory of Qinghai-Tibetan Plateau, Institute of Qinghai-Tibetan Plateau, Southwest Minzu University, Chengdu, China; ^2^Key Laboratory of Vegetation Restoration and Management of Degraded Ecosystems, South China Botanical Garden, Chinese Academy of Sciences, Guangzhou, China; ^3^Key Laboratory of Geospatial Technology for the Middle and Lower Yellow River Regions, Ministry of Education, College of Environment and Planning, Henan University, Kaifeng, China; ^4^Guangxi Key Laboratory of Forest Ecology and Conservation, State Key Laboratory for Conservation and Utilization of Agro-Bioresources, College of Forestry, Guangxi University, Nanning, China

**Keywords:** nitrogen deposition, canopy nitrogen addition, soil microbial community, bacteria and fungi, temperate forest, soil layer

## Abstract

Atmospheric nitrogen (N) deposition is known to alter soil microbial communities, but how canopy and understory N addition affects soil bacterial and fungal communities in different soil layers remains poorly understood. Conducting a 6-year canopy and understory N addition experiment in a temperate forest, we showed that soil bacterial and fungal communities in the organic layer exhibited different responses to N addition. The main effect of N addition decreased soil bacterial diversity and altered bacterial community composition in the organic layer, but not changed fungal diversity and community composition in all layers. Soil pH was the main factor that regulated the responses of soil bacterial diversity and community composition to N addition, whereas soil fungal diversity and community composition were mainly controlled by soil moisture and nutrient availability. In addition, compared with canopy N addition, the understory N addition had stronger effects on soil bacterial Shannon diversity and community composition but had a weaker effect on soil bacteria richness in the organic soil layer. Our study demonstrates that the bacterial communities in the organic soil layer were more sensitive than the fungal communities to canopy and understory N addition, and the conventional method of understory N addition might have skewed the effects of natural atmospheric N deposition on soil bacterial communities. This further emphasizes the importance of considering canopy processes in future N addition studies and simultaneously evaluating soil bacterial and fungal communities in response to global environmental changes.

## Introduction

Atmospheric nitrogen (N) deposition caused by anthropogenic activities has dramatically increased over the past century and is expected to increase continuously in future (Galloway et al., 2008; Stevens, 2019). As the temperate forest is generally considered to be limited by N (Hedin, 2004; Lebauer and Treseder, 2008), the increase in N deposition could relieve N limitation and stimulate plant growth (Thomas et al., 2010), increase litter biomass (Lebauer and Treseder, 2008), and thus enhance carbon (C) sequestration in temperate forests (Frey et al., 2014; Zak et al., 2017). Moreover, elevated N input can cause soil acidification (Lu et al., 2014), shift organic matter chemistry (Frey et al., 2014), and further alter soil microbial community composition (Lladó et al., 2017; Carrara et al., 2018). Soil microbial community plays an important role in soil organic matter decomposition, nutrient cycling, and other ecosystem functions and processes (Van Der Heijden et al., 2008; Crowther et al., 2019), and its responses to increasing N deposition may have profound consequences for global C and nutrient cycling and climate changes (Wang et al., 2018a). Therefore, understanding how soil microbial communities respond to elevated N deposition is beneficial to explicate the mechanisms of ecosystem C and nutrient cycling under N deposition, and it is important for predicting the feedback of terrestrial ecosystems to global climate changes.

Microbial diversity and community composition are key determinants of their ecological functions (Nannipieri et al., 2003; Philippot et al., 2013). It has been recognized that the elevated N deposition could influence soil microbial diversity and community composition (Freedman et al., 2015; Zhou et al., 2017; Dai et al., 2018; Liu et al., 2020b; He et al., 2021). For the bacterial community, most studies have shown that N addition generally reduces bacterial diversity and alters bacterial community composition (Freedman et al., 2015; Zeng et al., 2016; Nie et al., 2018; Wang et al., 2018c; Wu et al., 2019). For the fungal community, the diversity and community composition could be highly variable and inconsistent in response to N addition, as influenced by ecosystem types, soil conditions, duration of treatment, etc. (Mueller et al., 2014; Zhao et al., 2020). Some studies found that N addition could enhance or reduce fungal diversity and alter fungal community composition (Freedman et al., 2015; Kaspari et al., 2017; Chen et al., 2018; He et al., 2021), but others showed no effect of N addition on fungal diversity and community composition (Carrara et al., 2018; Li et al., 2019; Liu et al., 2021). Several environmental factors have been used to explain the responses of soil bacterial and fungal communities to N addition, such as soil pH and N availability (Rousk et al., 2010; Lladó et al., 2017; Nie et al., 2018; Wang et al., 2018a; Liu et al., 2021). Soil pH is widely recognized as an important predictor of soil bacterial communities under N addition (Rousk et al., 2010; Maestre et al., 2015; Fierer, 2017; Liu et al., 2021), whereas N availability is often thought to be a key regulator of soil fungal communities under N addition (Gilliam et al., 2011; Chen et al., 2018; He et al., 2021). To understand the implications of N deposition on soil ecosystem processes under future global change scenarios, general patterns and mechanisms in both bacterial and fungal communities in response to N deposition need to be investigated simultaneously (Baldrian et al., 2012; Romanowicz et al., 2016).

In forest ecosystems, many assessments of N deposition on soil microbial community are based on experiments of understory addition of N, that is, by directly adding N to the forest floor (Frey et al., 2004; Nie et al., 2018; Wang et al., 2018b). However, in reality, the most reactive N will be deposited first on the forest canopy, where it can be taken up by canopy tree leaves or immobilized by canopy biota before the residual N finally reaches the forest floor (Gaige et al., 2007; Houle et al., 2015). Field studies showed that forest canopies could intercept a large portion of N from atmospheric N deposition (Gaige et al., 2007; Dail et al., 2009; Liu et al., 2020a). A recent study in a temperate forest indicated that the forest canopy retained 52% and 44% of the N added to the forest canopy at the level of 25 kg N ha^−1^ year^−1^ and 50 kg N ha^−1^ year^−1^, respectively (Liu et al., 2020a). Using the canopy N budget method, Gaige et al. (2007) found that the canopy of mature coniferous forest intercepted more than 70% of the deposited N. A stable isotope tracer experiment found that ^15^${\text{N}-\text{NH}}_{4}^{+}$ deposited on the canopy could be directly taken up by leaves (Wang et al., 2021b). If most of the deposited N is utilized and retained by the forest canopy, it may mitigate the direct impact of N on understory plants and soil biota (Liu et al., 2020a). Therefore, previous studies that ignored the canopy processes might have overestimated the influences of N deposition on forest soil microbial communities. Although some field manipulative experiments have employed canopy N addition to simulating atmospheric N deposition in forest ecosystems (Gilliam and Hockenberry, 2006; Gaige et al., 2007; Dail et al., 2009; Gilliam et al., 2018), few studies have compared the effects of canopy N addition and understory N addition on forest ecosystem simultaneously. In addition, the effects of canopy and understory N addition on soil bacterial and fungal communities in the temperate forest are still not fully understood.

Here, we use a novel field manipulation experiment to compare the effects of canopy N addition (CN) with those of understory N addition (UN) on soil bacterial and fungal communities in different soil layers. The N addition experiment was conducted for a 6-year period in a temperate deciduous forest located in central China. Illumina Hiseq sequencing of bacterial 16S rRNA and fungal ITS genes was used to determine the diversity and composition of soil bacterial and fungal communities, respectively. We hypothesized that (1) N addition would decrease soil bacterial and fungal diversity and alter their community composition, especially bacterial communities; and (2) canopy N addition might have less significant effects on soil bacterial or fungal communities than understory N addition.

## Materials and Methods

### Study Site

This study was conducted at a field experimental station in a temperate forest, in the Jigongshan National Nature Reserve (31°46′-31°52' N, 114°01′-114°06′ E), Henan Province, China. The vegetation type at this site is a temperate deciduous broadleaf forest. Dominant canopy tree species include *Liquidambar formosana* Hance, *Quercus acutissima* Carruth, and *Quercus variabilis* Bl. The study site is within a transitional zone from a subtropical to a warm temperate climate region, with an average annual precipitation of 1,119 mm (80% of the precipitation from April to October) and an average annual temperature of 15.2°C (Zhang et al., 2015). The forest soil is a yellow-brown sandy-loam soil with a pH of 5.0–6.0. The background wet N deposition rate at this study site is approximately 19.6 kg N ha^−1^ year^−1^ (Zhang et al., 2015).

### Experimental Manipulation

This experiment contained two methods of N addition: understory N addition (UN) and canopy N addition (CN). For CN, we set up a forest canopy spraying system, with a 35-meter high tower in the center of each plot to support the sprinklers and pumps to deliver the N solutions to the forest canopy. For UN, we applied an automatic irrigation system, with the sprinklers installed 1.5 m above the ground. The experiment was set as a randomized block design with four blocks, and each block included five treatments: (1) control, without N addition (CT), (2) canopy N addition at 25 kg N ha^−1^ year^−1^ (CN25), (3) canopy N addition at 50 kg N ha^−1^ year^−1^ (CN50), (4) understory N addition at 25 kg N ha^−1^ year^−1^ (UN25), and (5) understory N addition at 50 kg N ha^−1^ year^−1^ (UN50). The dose of 25 kg N ha^−1^ year^−1^ was selected to simulate the background of N deposition at this study site. The dose of 50 kg N ha^−1^ year^−1^ was selected to simulate future atmospheric N deposition, and it also referred to the experience of other N deposition manipulation experiments. The N source in the treatments was NH_4_NO_3_. The NH_4_NO_3_ solution was sprayed monthly during the growing season from April to October. The total amount of water used for N addition per year was about 21 mm of precipitation, which was <1% of the total annual precipitation in this study site. More detailed information about the UN and CN system was described by Zhang et al. (2015).

### Sampling

Samples in the litter layer, organic soil layer, and mineral soil layer of each treatment plot were collected in July 2018. In each plot, we randomly selected eight spots and used a 20 cm × 20 cm frame to collect all the undecomposed and partially decomposed litter in the litter layer as litter layer samples. After litter layer removal, we collected the 0- to 2-cm thick humic soil in the same 20 cm × 20 cm frame below the litter layer as organic soil layer samples and then collected 0- to 10-cm mineral soil samples in the center of the 20 cm × 20 cm frame with a soil auger (38 mm in diameter). Eight samples of litter layer, organic soil layer, and mineral soil layer from the same treatment plot were mixed into one composite litter sample, organic soil sample, and mineral soil sample, respectively. A total of 60 samples were collected in this study (3 soil layers × 5 treatments × 4 blocks = 60). All the fresh samples were stored in iceboxes and transferred to the laboratory as soon as possible for further sample processing.

For litter samples, each fresh sample was mixed well and passed through a 2-mm sieve. Part of the fresh sample was cut into fine pieces, one was stored at −80°C for DNA extraction, and another one was stored at 4°C for the determination of the ammonium nitrogen (${\text{NH}}_{4}^{+}$), nitrate-nitrogen (${\text{NO}}_{3}^{-}$), and available P. The remaining fresh litter sample was oven-dried at 65°C and ground for chemical properties analysis. For organic soil and mineral soil samples, each fresh sample was mixed well and passed through a 2-mm sieve to remove roots and stones. Part of the fresh sample was stored at −80°C for DNA extraction, and another part was stored at 4°C for the ${\text{NH}}_{4}^{+}$, ${\text{NO}}_{3}^{-}$, and available P analysis. The remaining part was air-dried for the determination of soil chemical properties.

### Chemical Analysis

Soil moisture content was determined by the drying-weighing method. The pH of the organic and mineral soil samples was determined in a 1:2.5 soil/water suspension, and the pH of the litter sample was measured in a 1:10 litter/water suspension with a pH meter (Mettler Delta 340). Soil ${\text{NH}}_{4}^{+}$ and ${\text{NO}}_{3}^{-}$ were extracted with 2 mol L^−1^ KCl solution and then determined by a flow injection automatic analyzer (Lachat Instruments, Mequon, WI). Soil dissolved organic C (DOC) and total dissolved N (TDN) were extracted with 0.5 mol L^−1^ K_2_SO_4_ solution, and the concentrations of DOC and TDN in the extracts were determined by a TOC analyzer (TOC-5000, Shimadzu). Soil dissolved organic N (DON) was equal to TDN minus the sum of ${\text{NH}}_{4}^{+}$ and ${\text{NO}}_{3}^{-}$. Soil available P was first adsorbed by anion exchange resin membranes and then extracted by 0.5 mol L^−1^ dilute HCl, and the extracts were analyzed by the malachite green method (Veldhoven and Van Mannaerts, 1987). Total carbon (TC) and total N (TN) were measured by a C/N elemental analyzer (IsoPrime100, Isoprime).

### DNA Extraction and Illumina Sequencing

DNA was extracted from 0.5 g soil samples using the PowerSoil DNA Isolation Kit (MoBio Laboratories, Carlsbad, CA, USA) according to the manufacturer's instructions. The quantity and quality of the extracted DNA were examined using a Nanodrop Spectrophotometer (Thermo Fisher Scientific, Carlsbad, CA, USA). For bacterial communities, the V4–V5 hypervariable region of the 16S rRNA gene was amplified using the primer sets 515F (5′-GTGYCAGCMGCCGCGGTAA-3′) and 926R (5′-CCGYCAATTYMTTTRAGTTT-3′). For fungal communities, the ITS2 region was amplified using the primer sets ITS2-F (5′-GCATCGATGAAGAACGCAGC-3′) and ITS2-R (5′-TCCTCCGCTTATTGATATGC-3′). Polymerase chain reaction (PCR) amplification products were purified and recovered using a 1.8% (w/v) agarose gel electrophoresis method. The purified PCR products were sequenced using the Illumina HiSeq 2500 platform (Illumina, Inc., San Diego, CA, United States).

### Bioinformatic Analysis

The raw sequences were trimmed of reads containing ambiguous bases (<200 bp), barcodes, singletons, and long homopolymers to ensure high-quality data, and the high-quality paired-end reads were merged by USEARCH (V10.0.240) with minimal overlap of 16 bp. All filtered sequences were clustered into operational taxonomic units (OTUs) at the 97% similarity level (Edgar, 2013). Taxonomic annotations of each OTU were annotated using the Ribosomal Database Project Classifier tool. Representative sequences were annotated against the SILVA database (Quast et al., 2012) for bacteria and the UNITE database (Tedersoo et al., 2018) for fungi. A total of 4,451,886 and 4,393,727 high-quality Illumina sequencing reads were obtained for bacteria and fungi, respectively. All data were submitted to the Sequence Reads Archive (SRA) at the National Center for Biotechnology Information (NCBI) with accession numbers PRJNA801901 and PRJNA801925.

### Data Analyses

The OTU richness and Shannon diversity index calculated by Mothur (version v.1.30) were used as the metrics of bacterial and fungal α-diversity. Differences between the treatments were tested using a one-way analysis of variance (one-way ANOVA) followed by an *LSD* test. Spearman's correlation analysis was conducted to express the relationship between soil properties and microbial community variables, such as bacterial or fungal α-diversity. These analyses were mainly performed in SPSS 22.0 (SPSS, Chicago, IL, United States).

The multivariate permutational analysis of variance (PERMANOVA) was used to analyze the variances in bacterial and fungal community compositions and visualized by non-metric multidimensional scaling (NMDS). Adonis analysis was further performed to compare the significant differences among treatments. Redundancy analysis (RDA) through 999 Monte Carlo permutations was performed to determine the significant environmental factors contributing to bacterial and fungal community compositions. Prior to the analyses, variance inflation factors (VIF) were calculated to assess the multicollinearity of predictor variables. This led to the removal of soil C:N ratio, leaving soil moisture, pH, TC, TN, DOC, DON, ${\text{NH}}_{4}^{+}$, ${\text{NO}}_{3}^{-}$, and AP as predictor variables with low collinearity. The VIF values of these variables were shown in Supplementary Table S3. An RDA-based variance partitioning analysis (VPA) was conducted to assess the relative importance of soil pH, moisture, soil nutrients (TC and TN), and nutrient availability (DOC, DON, NH${\text{NH}}_{4}^{+}$, ${\text{NO}}_{3}^{-}$, and AP) in shaping bacterial and fungal community compositions. The statistical analyses in this part were performed in R software using the “vegan” package.

## Results

### Soil Properties

Understory N addition (UN) significantly increased soil moisture in the litter layer, but canopy N addition (CN) had no significant effects on it (Supplementary Table S1). Compared to the control (CT), both CN and UN treatments reduced soil pH in the organic soil layer, and the UN50 treatment decreased it in the mineral soil layer (*P*< *0.05*, Supplementary Table S1) as well. The CN25 and UN25 treatments reduced TC and TN contents in the organic layer (*P*< *0.05*, Supplementary Table S1). The concentration of ${\text{NH}}_{4}^{+}$ increased under both methods of N addition in the litter layer but decreased under the UN25 treatment in the mineral soil layer (*P*< *0.05*, Supplementary Table S1), while the concentration of ${\text{NO}}_{3}^{-}$ increased only under the UN50 treatment in the litter layer (*P*< *0.05*, Supplementary Table S1). The UN25 treatment reduced DOC concentration in the organic layer, but the CN50 treatment enhanced it in the mineral soil layer (*P*< *0.05*, Supplementary Table S1). There was no significant difference in AP and C:N ratio under both methods of N addition in all layers (*P* > *0.05*, Supplementary Table S1).

### Relative Abundance of Dominant Bacterial and Fungal Phyla

**Figure 1:**
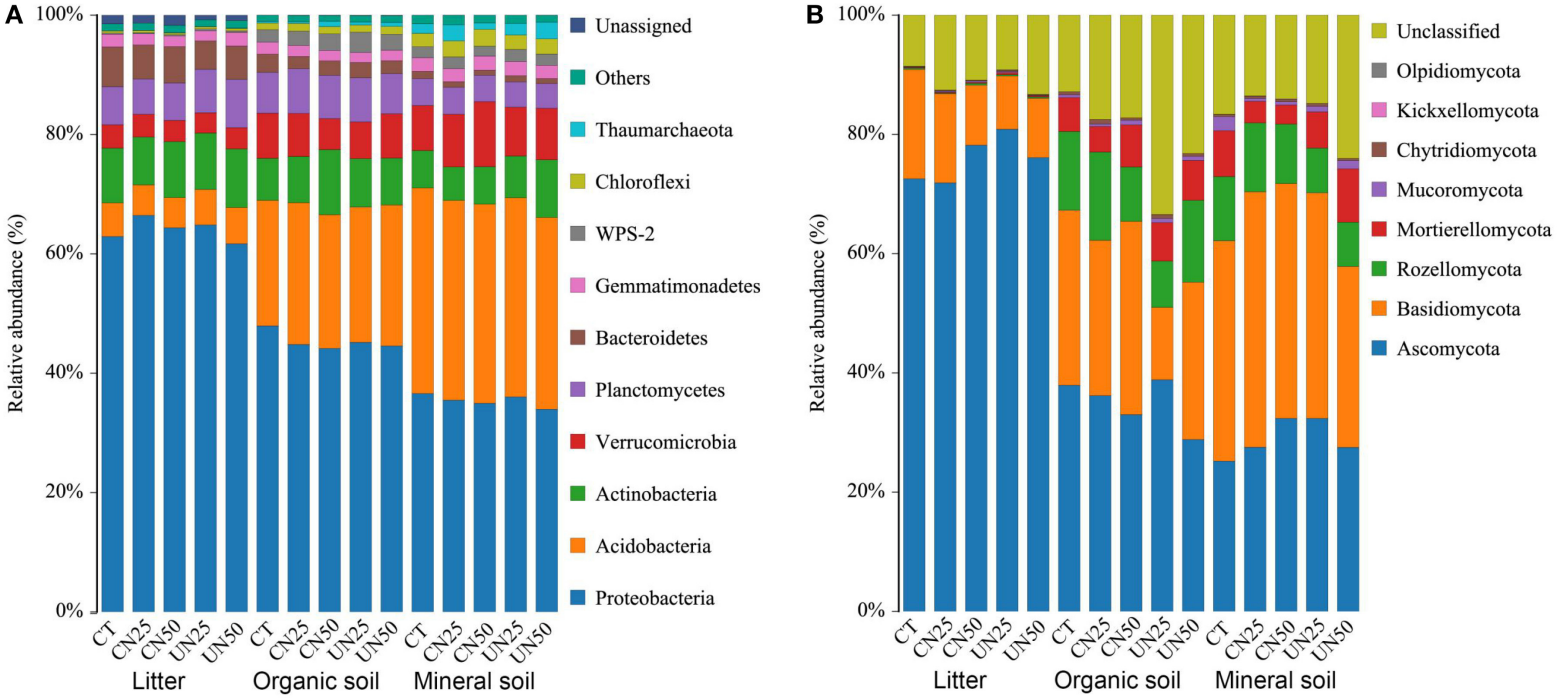
Relative abundance of the dominant bacterial groups **(A)** and fungal groups **(B)** at the phylum level under different N addition treatments and different soil layers. CT, control without N addition; CN25, canopy N addition with 25 kg N ha^−1^ year^−1^; CN50, canopy N addition with 50 kg N ha^−1^ year^−1^; UN25, understory N addition with 25 kg N ha^−1^ year^−1^; UN50, understory N addition with 50 kg N ha^−1^ year^−1^.

Proteobacteria, Acidobacteria, and Actinobacteria were the predominant bacterial phyla across all of the samples, which accounted for nearly 80% of the whole bacterial community (Figure 1A). In addition, Verrucomicrobia, Planctomycetes, Bacteroidetes, Gemmatimonadetes, and Chloroflexi were present at relatively low abundance. Ascomycota and Basidiomycota were the predominant fungal phyla across all of the samples, which accounted for 50–90% of the whole fungal community (Figure 1B). Rozellomycota and Mortierellomycota were the other two dominant fungal phyla at relatively low abundance.

N addition altered the relative abundance of some dominant bacterial phyla (Supplementary Figure S1), but did not alter the relative abundance of dominant fungal phyla (Supplementary Figure S2). Both canopy and understory N addition decreased the relative abundance of Proteobacteria in the organic layer (*P*< *0.05*, Supplementary Figure S1). Understory N addition also reduced the relative abundance of Bacteroidetes in all layers (*P*< *0.05*, Supplementary Figure S1f). However, the relative abundance of Verrucomicrobia was reduced in the organic layer and increased in the mineral layer only under the CN50 treatment (*P*< *0.05*, Supplementary Figure S1d). Compared to the control, the CN50 treatment increased the relative abundance of Actinobacteria in the organic layer, and the UN50 treatment increased it in the mineral layer (*P*< *0.05*, Supplementary Figure S1c). None of the dominant fungal phyla had significant responses to the canopy and understory N addition (*P* > *0.05*, Supplementary Figure S2).

### Diversity and Composition of Bacterial and Fungal Communities

**Figure 2:**
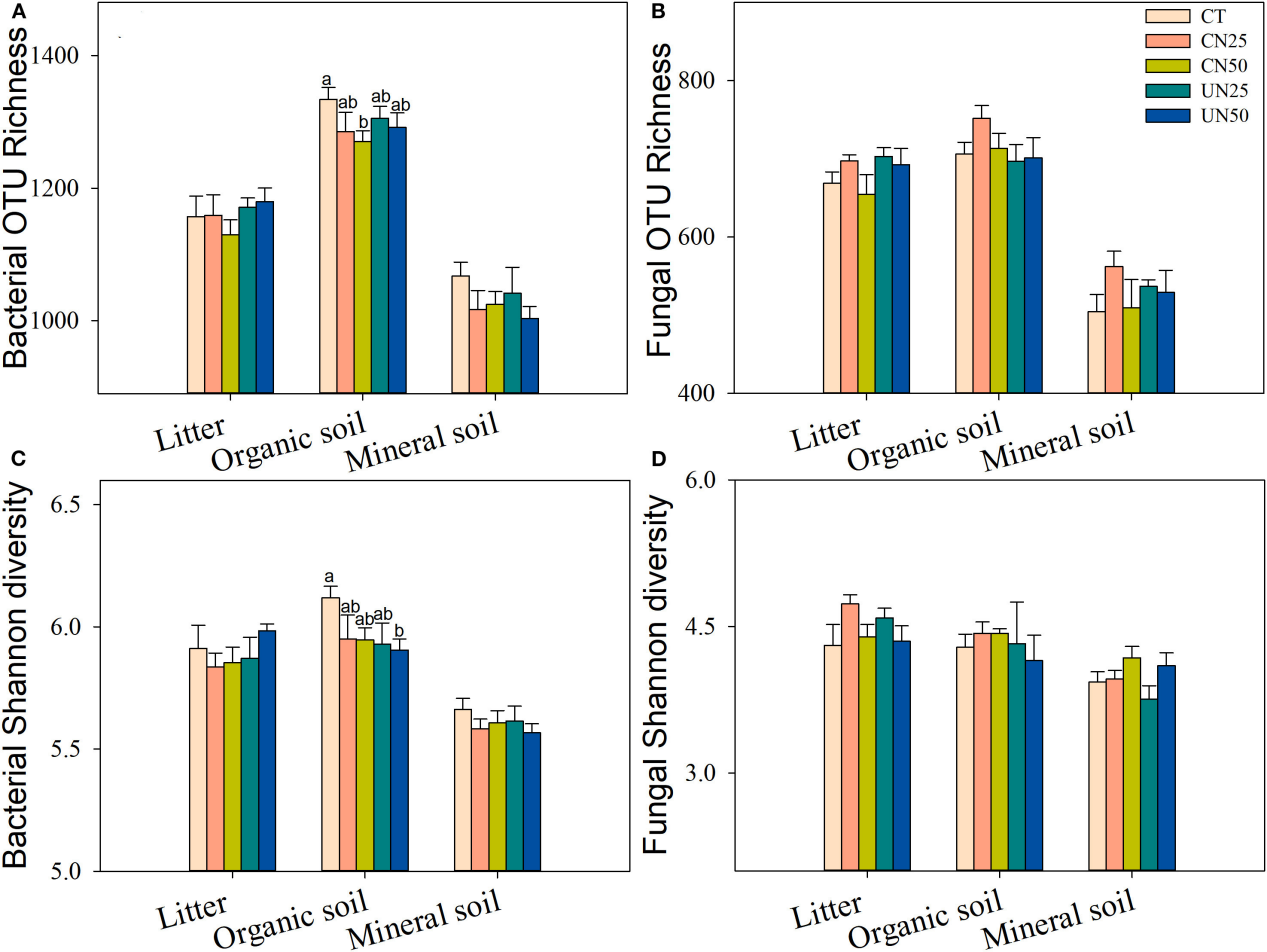
Effects of N addition treatments on soil bacterial **(A,C)** and fungal **(B,D)** alpha-diversity in different soil layers. Values are means ± standard error. Within each group of five treatments, values with different letters represent significant differences among the five treatments (*LSD* test, *P* < 0.05). CT, control without N addition; CN25, canopy N addition with 25 kg N ha^−1^ year^−1^; CN50, canopy N addition with 50 kg N ha^−1^ year^−1^; UN25, understory N addition with 25 kg N ha^−1^ year^−1^; UN50, understory N addition with 50 kg N ha^−1^ year^−1^.

**Figure 3:**
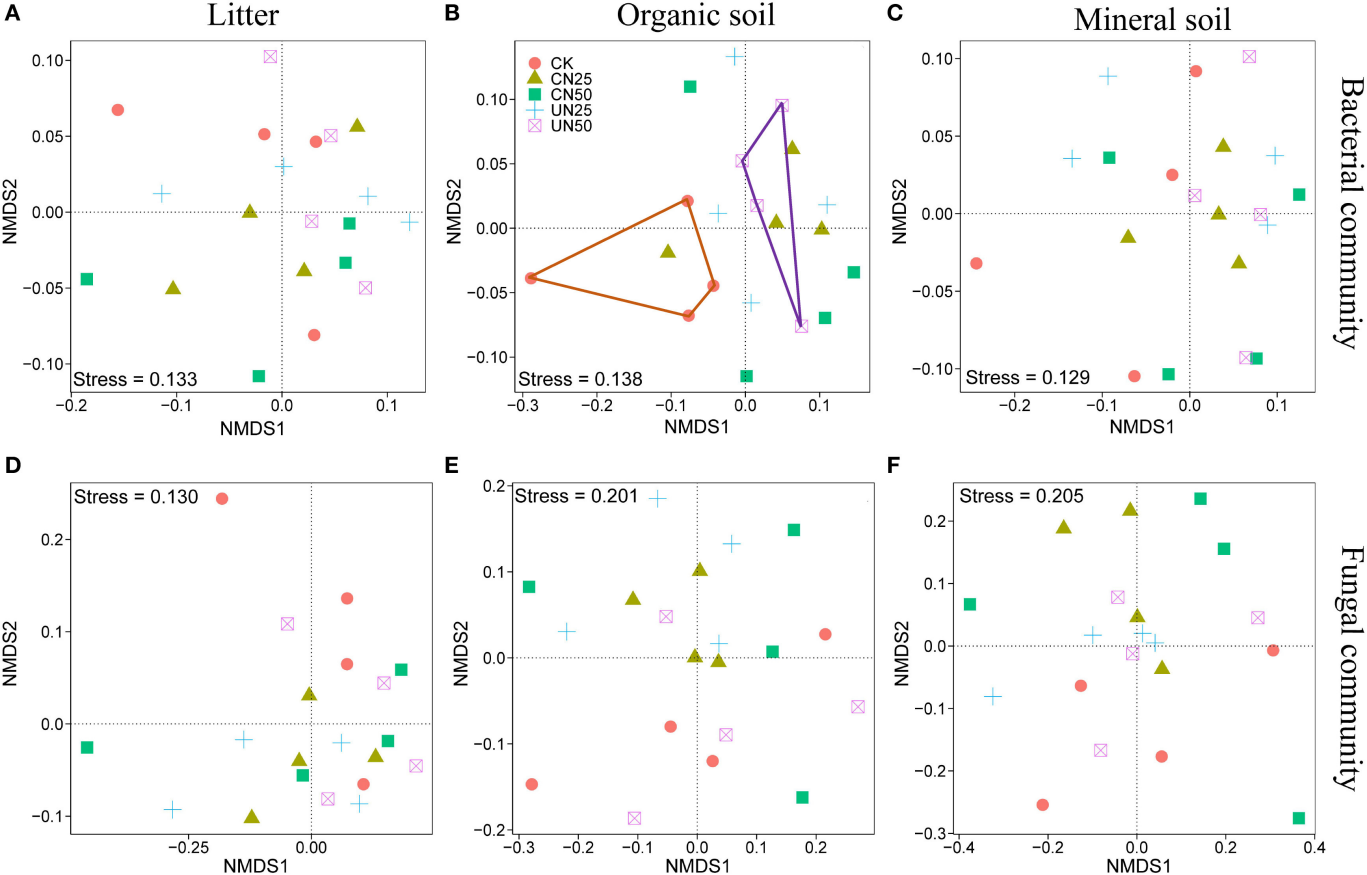
Nonmetric multidimensional scaling (NMDS) analysis based on the Bray–Curtis distance for the bacterial **(A–C)** and fungal **(D–F)** community compositions under different N addition treatments in different soil layers.

Bacterial and fungal α-diversity responded differently to the canopy and understory N addition (Figure 2). Canopy N addition decreased bacterial OTU richness in the organic layer, and understory N addition decreased bacterial Shannon diversity in this layer (*P*< *0.05*, Figures 2A,C). However, fungal α-diversity indexes (both OTU richness and Shannon diversity index) in all layers were unaffected by the canopy and understory N addition (*P* > *0.05*, Figures 2B,D). For community composition, understory N addition rather than canopy N addition significantly affected bacterial community composition in the organic layer, while both canopy and understory N addition had no significant effects on fungal community composition (Table 1). In addition, only the UN50 treatment was significantly different from the control for the bacterial community composition in the organic soil layer (Figure 3; Supplementary Table S2).

**Table 1:** Effects of canopy N addition (CN) and understory N addition (UN) on bacterial and fungal community compositions at OTU level based on Bray–Curtis distance, as determined by PERMANOVA.

	**Bacterial communities**				**Fungal communities**			
	**CN**		**UN**		**CN**		**UN**	
	**R^**2**^**	***P***	**R^**2**^**	***P***	**R^**2**^**	***P***	**R^**2**^**	***P***
Litter	0.15	0.72	0.16	0.62	0.16	0.71	0.15	0.84
Organic soil	0.24	0.17	0.28	**0.01**	0.14	0.97	0.19	0.26
Mineral soil	0.20	0.33	0.16	0.61	0.17	0.60	0.15	0.91

*P-values reflecting statistical significance are shown in boldface (P < 0.05)*.

### Relationships Between Microbial Communities and Soil Properties

**Figure 4:**
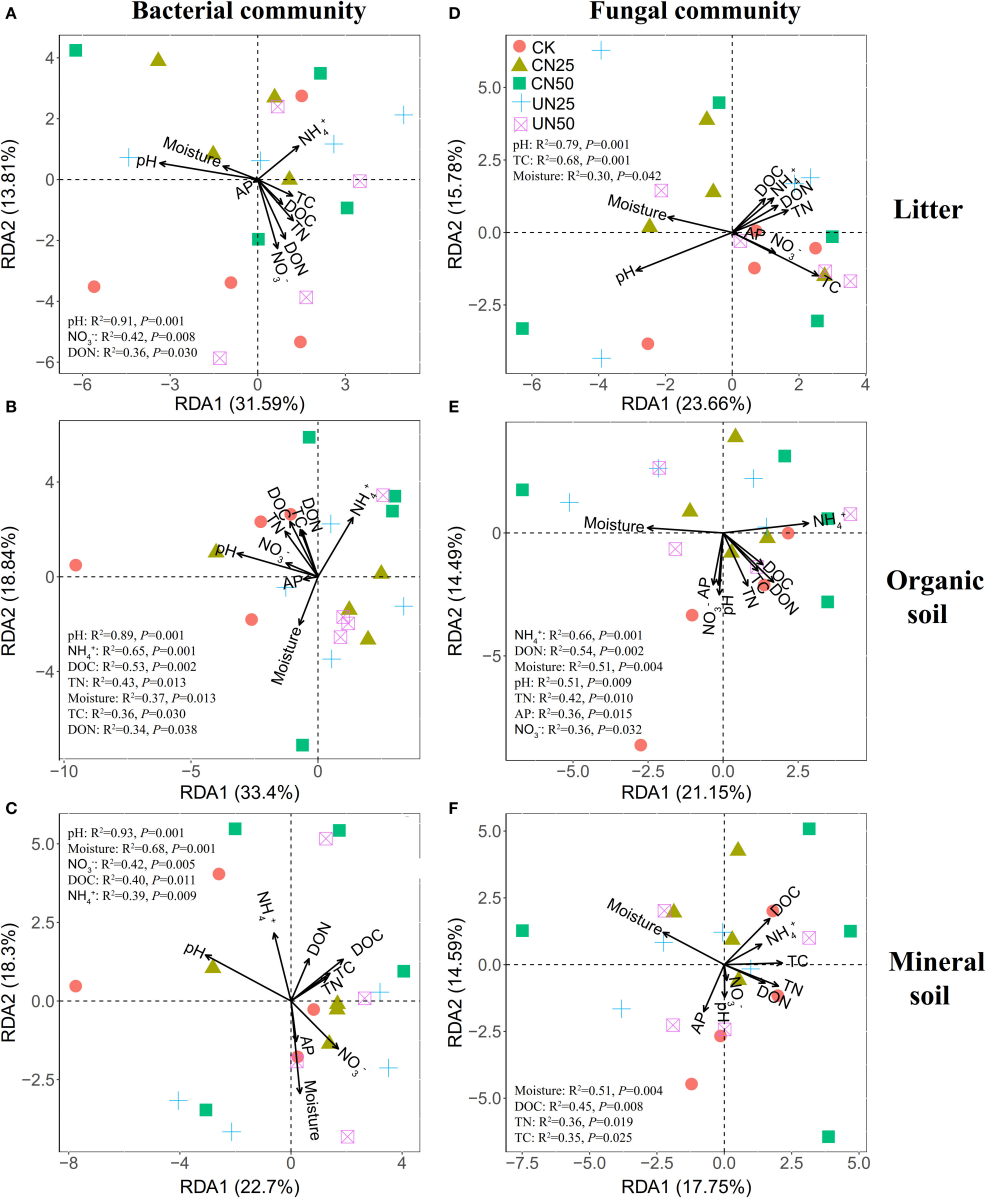
Redundancy analysis (RDA) of bacterial **(A–C)** and fungal **(D–F)** community compositions under different N addition treatments in different soil layers. Significant influencing factors derived from Monte Carlo tests are shown (*P* < 0.05).

**Figure 5:**
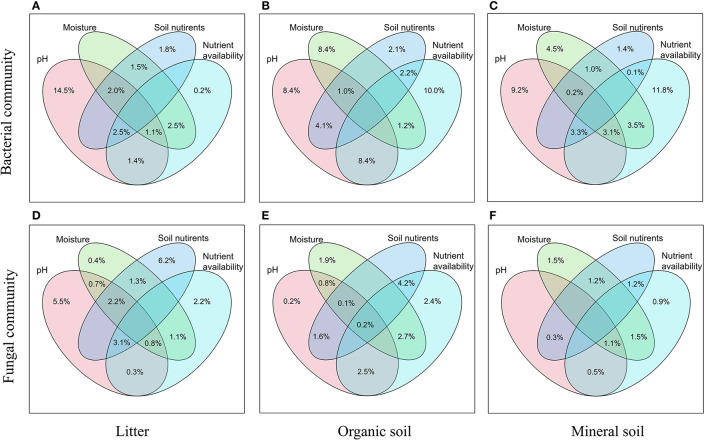
Relative importance of different predictors of bacterial **(A–C)** and fungal **(D–F)** communities in different soil layers was revealed by RDA-based VPA analysis. Soil nutrients contain TC and TN, and nutrient availability contains DOC, DON, ${\text{NH}}_{4}^{+}$, ${\text{NO}}_{3}^{-}$, and AP.

Bacterial α-diversity (both OTU richness and Shannon diversity index) was mainly positively related to soil pH in the organic soil and mineral soil layers, whereas fungal α-diversity was mainly positively related to soil nutrient availability (e.g., DOC in the litter layer, DON, and AP in the mineral layer) (*P* < 0.05, Table 2). Redundancy analysis (RDA) showed that soil pH was the most important factor for shaping the bacterial community composition in all layers, which had the strongest r^2^ and the lowest *P*-values (*P* < 0.05, Figures 4A–C). Soil moisture, TC, TN, ${\text{NH}}_{4}^{+}$, DOC, and DON also had significant influences on bacterial community composition in the organic soil layer (*P* < 0.05, Figure 4B). Soil ${\text{NH}}_{4}^{+}$ and soil moisture were the most important factors (based on *r*^2^ and *P*-values) that influenced the fungal community composition in the organic soil layer and mineral soil layer, respectively (*P* < 0.05, Figures 4E,F). Other soil nutrients (TC and TN) and nutrient availability (DOC, DON, ${\text{NO}}_{3}^{-}$, and AP) also had significant effects on fungal community composition (*P* < 0.05, Figures 4D–F). The RDA-based VPA analysis showed that soil pH explained the main proportion of the bacterial community variations in all the layers (Figures 5A–C), and soil nutrient availability also explained large proportions of the bacterial community variations in the organic soil and mineral soil layers (Figures 5B,C). However, for the fungal community, soil nutrients, nutrient availability, and moisture explained the main proportion of the variations in the litter layer, organic soil layer, and mineral soil layer, respectively (Figures 5D–F).

**Table 2:** Spearman's correlations between soil properties and the OTU richness and Shannon diversity of bacterial and fungal communities in different soil layers.

	**Bacterial communities**						**Fungal communities**					
	**Litter**		**Organic soil**		**Mineral soil**		**Litter**		**Organic soil**		**Mineral soil**	
	**Richness**	**Diversity**	**Richness**	**Diversity**	**Richness**	**Diversity**	**Richness**	**Diversity**	**Richness**	**Diversity**	**Richness**	**Diversity**
Moisture	0.17	−0.04	0.13	0.05	0.08	−0.03	0.33	0.26	0.06	0.24	0.14	0.42
pH	−0.17	−0.33	0.46*	0.69**	0.65**	0.84**	−0.18	−0.05	−0.01	−0.18	0.15	−0.01
TC	0.14	0.35	−0.22	−0.1	−0.32	−0.37	−0.16	−0.13	−0.11	−0.02	−0.41	0.05
TN	−0.05	−0.06	−0.06	0.07	−0.32	−0.47*	0.06	0.12	−0.24	−0.22	−0.25	0.16
C:N	0.06	0.17	−0.51*	−0.38	−0.38	−0.36	−0.17	−0.22	−0.11	0.04	−0.48*	−0.25
${\text{NH}}_{4}^{+}$	0.05	−0.01	−0.3	−0.17	−0.03	0.11	0.24	0.3	−0.01	−0.3	−0.36	−0.25
${\text{NO}}_{3}^{-}$	0.2	0.26	0.12	−0.04	−0.33	−0.54*	0.06	−0.15	0.03	0.1	−0.11	0.21
DOC	0.05	0.14	0	0.18	−0.05	−0.2	0.44	0.46*	−0.17	−0.15	0.17	0.23
DON	0	0.14	−0.13	−0.08	−0.23	−0.2	0.22	0.32	−0.09	−0.14	−0.12	0.50*
AP	−0.02	0.06	0.17	0	−0.03	−0.2	0.04	0.17	0.02	−0.14	0	0.47*

*P < 0.05; **P < 0.01. Significant *P* values (*P* < 0.05) are highlighted in bold.

*Richness: OTU richness; Diversity: Shannon diversity; TC, total carbon; TN, total nitrogen; C:N, the molar ratio of total carbon to total nitrogen; DOC, dissolved organic carbon; DON, dissolved organic nitrogen; AP, available phosphorus*.

## Discussion

### Different Effects of N Addition on Soil Bacterial and Fungal Communities

In support of our first hypothesis, we found that N addition decreased soil bacterial diversity and altered bacterial community composition in the organic soil layer (Figures 2, 3; Table 1), which is consistent with the results of many previous studies (Freedman et al., 2015; Zeng et al., 2016; Nie et al., 2018; Wang et al., 2018a; Wu et al., 2019). The reduction in bacterial diversity and alternation in bacterial community composition could be attributed to decreased soil pH caused by N addition. Soil pH was widely considered to be an important factor in regulating the alteration of soil bacterial diversity and community composition (Maestre et al., 2015; Fierer, 2017; Liu et al., 2021). Since most bacteria grow best around neutral pH, N-induced soil acidification has often been found to reduce bacterial diversity (Rousk et al., 2010; Nie et al., 2018). In addition, the reduced soil pH could cause high aluminum concentration (${\text{Al}}_{3}^{+}$) and trigger aluminum toxicity (Lu et al., 2014; Liu et al., 2021), which can be harmful to bacterial growth and thus change the bacterial community composition (Wang et al., 2018b, 2021a). In our experiment, soil pH was reduced substantially after 6-year N addition in the organic layer (Supplementary Table S1), and it was significantly correlated with bacterial diversity and community composition and explained a large proportion of the bacterial community variations in this layer (*P* < 0.05, Table 2; Figures 4, 5). These results indicate that soil pH is a key driving factor for the responses of bacterial diversity and community composition to N addition in the organic soil layer. Other studies believe that N availability (such as ${\text{NH}}_{4}^{+}$ concentration) is the main regulator of the alternations in soil bacterial diversity and community composition under N addition (Zeng et al., 2016; Nie et al., 2018). In this study, we found that the available N concentration (${\text{NH}}_{4}^{+}$ and ${\text{NO}}_{3}^{-}$) was not significantly correlated with soil bacterial diversity in the organic layer (*P* > *0.05*, Table 2), indicating organic soil bacterial diversity in this study site is mainly controlled by soil pH rather than N availability. Nevertheless, organic soil bacterial community composition was significantly correlated with available N concentration (${\text{NH}}_{4}^{+}$ and DON) and other environmental factors (such as soil moisture, TN, and DOC) (*P*< *0.05*, Figure 4B), and soil moisture and nutrient availability explained large proportions of the bacterial community variations in the organic soil (Figure 5B). These results suggest that the response of organic soil bacterial community composition to N addition is regulated by comprehensive environmental factors (e.g., soil pH, soil moisture, and nutrient availability).

However, contrary to our first hypothesis, soil fungal diversity and community composition in all layers exhibited no significant responses to N addition (Figures 2, 3; Table 1), suggesting fungal communities are highly adapted to elevated N deposition at our study site. This result differs from that of bacterial communities in the organic soil layer, indicating bacterial communities are more sensitive to N addition than the fungal communities in this layer. A recent study also reported that N input alters soil bacterial diversity but not fugal diversity in a grassland (Liu et al., 2021). This pattern may be contributed to the following three reasons. First, fungi exhibit wider pH ranges for optimal growth than bacteria (Rousk et al., 2010); thus, fungi tend to be more tolerant to soil acidification (Rousk et al., 2010; Herold et al., 2012). This relative insensitivity to changes in soil pH may be the reason why fungal communities remained constant after N addition in our experiment. Our study also found no significant correlation between soil pH and fungal diversity in all layers (*P* > *0.05*, Table 2), and soil pH just explained a little variation of the fungal community in the organic soil and mineral soil layers (Figures 5E,F). Second, compared to fungi, bacteria have a shorter turnover time and can respond more quickly to environmental changes (Yin et al., 2010; Li et al., 2019); thus, bacterial communities are more sensitive to N addition than fungal communities in this study. Third, compared to bacteria, fungal communities are often thought to be even more strongly coupled with plant community (van der Linde et al., 2018; Liu et al., 2021) and soil nutrients (Gilliam et al., 2011; Chen et al., 2018; He et al., 2021). In this study, we identified fungal diversity and community composition were closely related to soil moisture and nutrient availability (such as DOC, DON, AP, ${\text{NH}}_{4}^{+}$, and ${\text{NO}}_{3}^{-}$) (*P*< *0.05*, Table 2; Figure 4), and soil nutrients, nutrient availability, and soil moisture explained the main variations of fungal community composition in the litter layer, organic soil layer, and mineral soil layer, respectively (Figures 5D–F), indicating that the fungal community was mainly regulated by soil moisture and nutrient availability. Other studies revealed that the fungal community was mainly controlled by the plant community due to their greater reliance on plants for substrates and habitats (van der Linde et al., 2018; Liu et al., 2021). Unfortunately, we did not quantify plant community in this study, which precludes us from directly assessing the contribution of plant community to soil fungal communities. Further research on this issue needs to be considered to better understand the role of the plant community in regulating fungal communities under N deposition in this studied forest.

In addition, the effects of N addition on soil bacterial communities varied with soil layers. In this study, N addition just reduced bacterial diversity and shifted bacterial community composition only in the organic soil layer, but not in the litter layer and the mineral soil layer (Figures 2, 3; Table 1), suggesting the layer dependence of the N deposition controls on bacterial communities. This result in line with our previous study reported that soil enzyme kinetics responses to N addition differed in the litter, organic soil, and mineral soil layers (Liu et al., 2020c). Theoretically, the amount of added N will gradually decrease as the soil layer deepens; thus, the amount of N entering the litter layer, organic soil layer, and mineral soil layer are different, which may affect the soil microbial communities differently. The litter layer is in the uppermost and has large porosity, most of the added N would easily be leached to the organic soil layer below the litter layer, and thus, the response of the organic soil layer is more sensitive to N addition than the litter layer. In addition, due to the interception of the organic layer, less added N may reach the mineral soil layer, so the response of the mineral soil layer is less sensitive than the organic layer. This may be an important reason for the different results among different soil layers. A recent study also found that the climatic controls on soil microbial community composition varied with soil horizons (Dove et al., 2021). This finding indicates that the results of the responses of soil bacterial communities to N deposition could be biased if only a single soil layer is investigated. For a more comprehensive understanding of the effects of N deposition on forest soil bacterial communities, more than one sampling forest soil layer should be considered in future studies.

### Different Effects of Canopy and Understory N Addition on Microbial Communities

In this study, we observed that understory N addition significantly decreased the bacterial Shannon diversity and significantly altered bacterial community composition in the organic soil layer, but canopy N addition had no significant effects on bacterial Shannon diversity and community composition in this layer (Figures 2, 3; Table 1), which was consistent with our second hypothesis that canopy N addition has less significant effects on soil bacterial communities than understory N addition. There were at least three reasons for this phenomenon. First, it could be explained by the large proportion of the added N intercepted by the forest canopy with canopy N addition (Liu et al., 2020a). A recent study in our studied forest has reported that the canopy processes have retained 44 and 52% of the N added to the forest canopy by CN50 and CN25, respectively (Liu et al., 2020a). Other previous studies have also revealed that the forest canopies could retain a substantial portion of the deposited N (Gaige et al., 2007; Dail et al., 2009). Thereby canopy N addition mitigated the direct effects of N addition on soil bacterial communities.

Second, it could be attributable to the different influence ways of understory N addition and canopy N addition on soil bacterial communities in forest ecosystems. The method of understory N addition is to add N solution directly onto the forest floor, which could influence soil microbial communities directly (Shi et al., 2016). By contrast, the method of canopy N addition is sprayed N solution onto the forest canopy, and the N deposited on the canopy could be uptake by tree leaves, twigs, and branches (Adriaenssens et al., 2012; Houle et al., 2015; Wang et al., 2021b). Therefore, canopy N addition would directly influence plants first and then indirectly influence soil microbial communities. The plant community is known to influence soil bacterial diversity and composition (Wardle et al., 2004; Liu et al., 2020b). Canopy N addition may influence soil bacterial communities indirectly through altering plant leaf, litter, and root traits of the canopy trees. A recent study in our studied forest found that canopy N addition increased the leaf N content of canopy trees more than understory N application (Li et al., 2021), thereby enhancing the leaf photosynthesis (Wang et al., 2021b), which in turn would lead to an increase in the allocation of photosynthates to the belowground (Farrar and Jones, 2000; Hendricks et al., 2000). This process may have positive effects on soil microbial communities due to the photosynthates being the carbon source for microbial growth. Li et al. (2021) also found that the fine root biomass and production were significantly higher with canopy N addition than with understory N addition, and the enhanced fine root biomass and production may also have positive effects on soil microbial communities due to they can secrete more root exudates for microbial growth. Besides, as discussed earlier, soil pH had negative effects on soil bacterial communities (in section Different Effects of N Addition on Soil Bacterial and Fungal Communities). Therefore, organic soil bacterial communities were not significantly affected by canopy N addition which may be due to the tradeoff between positive effects from plant processes and negative effects from soil pH. However, understory N addition did not experience the canopy N retention processes; thus, its negative effects on bacterial communities mainly come from the soil pH. In addition, a previous study conducted in the same forest did not detect any significant changes in the leaf litter quantity and quality between canopy N addition and the control (Liu et al., 2020a). Similarly, our study also observed no significant effects of canopy N addition on litter C:N ratio (Supplementary Table S1). These results together indicate that the indirect influence of canopy N addition through litter is very small, so canopy N addition had weaker effects on soil bacterial communities than understory N addition did.

Another possible explanation might be that understory N addition caused more significant changes in soil chemical properties (e.g., moisture, pH, total and available C and N content) than canopy N addition did (Supplementary Table S1), while these soil properties can influence soil microbial communities directly or indirectly (Rousk et al., 2010; Lladó et al., 2017; Chen et al., 2018; Nie et al., 2018; Li et al., 2019) and consequently exaggerated its influences on soil bacterial communities. Therefore, our results suggest that the conventional method of understory N addition may inaccurately estimate the effects of atmospheric N deposition on soil bacterial communities, while the novel method of canopy N addition is a more realistic simulation of N deposition and the canopy process should be taken into account in future N deposition studies on forest ecosystems.

## Conclusion

In summary, our study found that N addition altered organic soil bacterial communities but not fungal communities in the studied forest, suggesting that bacterial communities in the organic soil layer are more sensitive to elevated N deposition than fungal communities at this study site. These divergent responses of bacterial and fungal communities to increased N input were driven by different mechanisms. The bacterial diversity and community composition were mainly regulated by soil pH, whereas the fungal diversity and community composition were mainly controlled by soil moisture and nutrient availability. In addition, canopy N addition had less significant effects on soil bacterial communities than understory N addition. Our results revealed that the effects of atmospheric N deposition on soil bacterial communities in forest ecosystems may be skewed by using the traditional understory N addition method because the important role of canopy processes was overlooked. We emphasize that the canopy processes need to be considered in future N deposition studies.

## Data Availability Statement

The datasets presented in this study can be found in online repositories. The names of the repositories and accession numbers can be found below: https://www.ncbi.nlm.nih.gov/bioproject/PRJNA801901; https://www.ncbi.nlm.nih.gov/bioproject/PRJNA801925.

## Author Contributions

SF and WS designed the experiment. YL performed the experiments, analyzed the data, and wrote the first draft of the manuscript. YL, XT, SF, and WS revised the manuscript. All authors contributed to the article and approved the submitted version.

## Funding

This study was supported by the Junwu Scholarship of Guangxi University (No. A3360051012), the National Natural Science Foundation of China (No. 31290222, 31130011, and 31425005), the Guangdong Province Baiqianwan Talents Program, the National Ten Thousand Talents Program, and the Southwest Minzu University Research Startup Funds (No. RQD2022020).

## Conflict of Interest

The authors declare that the research was conducted in the absence of any commercial or financial relationships that could be construed as a potential conflict of interest. The Reviewer KX declared a shared affiliation with the author XT at the time of the review.

## Publisher's Note

All claims expressed in this article are solely those of the authors and do not necessarily represent those of their affiliated organizations, or those of the publisher, the editors and the reviewers. Any product that may be evaluated in this article, or claim that may be made by its manufacturer, is not guaranteed or endorsed by the publisher.
