# Supplementary material for: Canopy and Understory Nitrogen Addition Alters Organic Soil Bacterial Communities but Not Fungal Communities in a Temperate Forest
Source: Front Microbiol. 2022 Jun 10;13:888121. doi: 10.3389/fmicb.2022.888121 (PMC9226683; doi:10.3389/fmicb.2022.888121)
Supplement: Supplementary file 1 [file Table_1.docx]

**Canopy and understory nitrogen addition alters organic soil bacterial communities but not fungal communities in a temperate forest**

***Yang Liu^1,2^, Xiangping Tan^2^, Shenglei Fu^3^, Weijun Shen^4^****

*^1^ Ecological Conservation and Restoration Laboratory of Qinghai-Tibetan Plateau, Institute of Qinghai-Tibetan Plateau, Southwest Minzu University, Chengdu, China*

*^2^ Key Laboratory of Vegetation Restoration and Management of Degraded Ecosystems, South China Botanical Garden, Chinese Academy of Sciences, Guangzhou, China*

*^3^ Key Laboratory of Geospatial Technology for the Middle and Lower Yellow River Regions, Ministry of Education, College of Environment and Planning, Henan University, Kaifeng, China*

*^4^ Guangxi Key Laboratory of Forest Ecology and Conservation, State Key Laboratory for Conservation and Utilization of Agro-bioresources, College of Forestry, Guangxi University, Nanning, Guangxi 530004, China*

*Correspondence author:

Weijun Shen (shenweijun@gxu.edu.cn)

College of Forestry, Guangxi University

100 Daxue Rd., Xixiangtang District

Nanning, Guangxi 530004, China

Tel/ Fax: (+86) 0771- 3271428

**Supplementary data**

**Table S1** The basic soil properties respond to canopy and understory N addition in different soil layers in the Jigongshan temperate forest. Values are means ± standard error. Within each group of five treatments, values with different letters represent significant differences among the five treatments (*LSD* test, *P* < 0.05). CT, control without N addition; CN25, canopy N addition with 25 kg N ha^−1^ yr^−1^; CN50, canopy N addition with 50 kg N ha^−1^ yr^−1^; UN25, understory N addition with 25 kg N ha^−1^ yr^−1^; UN50, understory N addition with 50 kg N ha^−1^ yr^−1^.

|  | Moisture% | pH | TC % | TN % | C:N | DOC mg kg^-1^ | DON mg kg^-1^ | NH_4_^+^ mg kg^-1^ | NO_3_^−^ mg kg^-1^ | AP mg kg^-1^ |  |
| --- | --- | --- | --- | --- | --- | --- | --- | --- | --- | --- | --- |
| Litter |  |  |  |  |  |  |  |  |  |  |  |
| CT | 26.5±0.8 b | 5.61±0.11 | 42.1±0.6 | 1.89±0.04ab | 26.1±0.7 | 6122.1±251.1ab | 1016.5±22.5 | 315.0±60.7c | 85.7±22.0b | 163.7±10.5ab |  |
| CN25 | 39.1±5.7 ab | 5.60±0.05 | 41.6±0.3 | 1.92±0.02a | 25.3±0.2 | 7312.3±413.1a | 1034.6±68.1 | 658.5±20.9a | 106.3±31.6b | 206.8±12.6a |  |
| CN50 | 34.6±6.9 ab | 5.51±0.13 | 41.4±0.6 | 1.87±0.08ab | 25.9±1.0 | 5322.6±485.4b | 897.1±105.8 | 572.2±62.0ab | 157.4±44.3ab | 182.9±8.0ab |  |
| UN25 | 39.7±6.1 ab | 5.50±0.10 | 40.9±0.5 | 1.75±0.06b | 27.3±1.0 | 5557.9±480.6b | 856.3±62.0 | 404.2±82.9bc | 82.3±11.3b | 148.5±18.6b |  |
| UN50 | 55.6±16.2 a | 5.45±0.06 | 42.0±0.2 | 1.91±0.05ab | 25.9±0.7 | 6247.3±781.5ab | 975.0±115.0 | 638.3±38.4a | 203.3±34.1a | 194.9±23.7ab |  |
| Organic soil | |  |  |  |  |  |  |  |  |  |  |
| CT | 26.3±3.3 | 4.62±0.17a | 13.3±1.5a | 0.8±0.07a | 19.5±1.1 | 429.1±53.6a | 75.0±11.3a | 27.6±6.5 | 67.1±11.3 | 23.6±4.4 |  |
| CN25 | 24.1±1.4 | 4.31±0.09b | 9.7±0.5b | 0.6±0.03b | 19.1±0.6 | 330.5±22.2ab | 52.4±5.3ab | 25.6±2.7 | 53.7±1.1 | 16.9±3.6 |  |
| CN50 | 25.9±4.0 | 4.17±0.05b | 12.3±1.6ab | 0.73±0.08ab | 19.6±1.5 | 401.3±47.3ab | 63.01±11.4ab | 34.3±7.1 | 58.6±5.8 | 24.2±2.2 |  |
| UN25 | 25.2±2.9 | 4.21±0.06b | 9.7±0.7b | 0.59±0.05b | 19.2±0.2 | 306.1±16.2b | 44.0±5.7b | 19.5±4.1 | 52.0±3.7 | 18.8±2.6 |  |
| UN50 | 30.0±5.3 | 4.15±0.03b | 10.7±1.1ab | 0.67±0.06ab | 18.4±0.5 | 376.1±15.1ab | 61.9±3.8ab | 30.4±7.0 | 67.7±7.8 | 23.9±3.7 |  |
| Mineral soil | |  |  |  |  |  |  |  |  |  |  |
| CT | 14.4±1.8 | 4.25±0.10a | 6.3±1.3 | 0.34±0.05 | 21.7±2.4 | 142.3±13.4b | 21.2±1.8ab | 3.6±0.9a | 11.8±2.4 | 6.9±2.0 |  |
| CN25 | 16.4±0.5 | 4.11±0.04ab | 5.1±0.5 | 0.3±0.02 | 20.0±1.2 | 165.1±9.4ab | 21.1±1.3ab | 3.0±0.4ab | 11.4±1.3 | 6.0±1.9 |  |
| CN50 | 14.8±3.0 | 4.12±0.08ab | 6.3±1.1 | 0.35±0.03 | 21.1±2.3 | 188.7±17.3a | 22.8±1.2ab | 2.7±0.2ab | 11.6±1.0 | 8.2±1.5 |  |
| UN25 | 17.4±2.1 | 4.06±0.05ab | 4.8±0.6 | 0.27±0.02 | 20.4±1.0 | 148.8±11.2b | 18.9±1.7b | 1.6±0.5b | 14.0±0.7 | 6.1±1.4 |  |
| UN50 | 17.3±2.9 | 4.03±0.04b | 5.3±0.8 | 0.31±0.04 | 19.7±0.9 | 165.7±7.1ab | 23.9±1.4a | 2.2±0.8ab | 15.2±1.6 | 10.8±4.2 |  |

Note: TC, total carbon; TN, total nitrogen; C:N, the molar ratio of total carbon to total nitrogen; DOC, dissolved organic carbon; DON, dissolved organic nitrogen; AP, available phosphorus.

**Table S2** Results of Adonis analysis to compare the differences in OTU composition among the canopy N addition (CN) and understory N addition (UN) treatments. P-values reflecting statistical significance are shown in boldface. CT, control without N addition; CN25, canopy N addition with 25 kg N ha^−1^ yr^−1^; CN50, canopy N addition with 50 kg N ha^−1^ yr^−1^; UN25, understory N addition with 25 kg N ha^−1^ yr^−1^; UN50, understory N addition with 50 kg N ha^−1^ yr^−1^.

|  |  |  | Bacterial communities | |  | Fungal communities | |
| --- | --- | --- | --- | --- | --- | --- | --- |
|  |  |  | R^2^ | *P* |  | R^2^ | *P* |
| Litter | CN | CT/CN25 | 0.13 | 0.21 |  | 0.11 | 0.28 |
|  |  | CT/CN50 | 0.10 | 0.33 |  | 0.11 | 0.29 |
|  |  | CN25/CN50 | 0.09 | 0.81 |  | 0.14 | 0.46 |
|  |  |  |  |  |  |  |  |
|  | UN | CT/UN25 | 0.13 | 0.18 |  | 0.09 | 0.40 |
|  |  | CT/UN50 | 0.11 | 0.25 |  | 0.11 | 0.27 |
|  |  | UN25/UN50 | 0.10 | 0.83 |  | 0.12 | 0.59 |
|  |  |  |  |  |  |  |  |
| Organic soil | CN | CT/CN25 | 0.17 | 0.08 |  | 0.10 | 0.39 |
|  |  | CT/CN50 | 0.20 | 0.07 |  | 0.10 | 0.34 |
|  |  | CN25/CN50 | 0.12 | 0.61 |  | 0.11 | 0.88 |
|  |  |  |  |  |  |  |  |
|  | UN | CT/UN25 | 0.20 | 0.07 |  | 0.14 | 0.11 |
|  |  | CT/UN50 | 0.24 | **0.036** |  | 0.14 | 0.17 |
|  |  | UN25/UN50 | 0.13 | 0.66 |  | 0.14 | 0.51 |
|  |  |  |  |  |  |  |  |
| Mineral soil | CN | CT/CN25 | 0.12 | 0.26 |  | 0.14 | 0.11 |
|  |  | CT/CN50 | 0.13 | 0.17 |  | 0.11 | 0.24 |
|  |  | CN25/CN50 | 0.17 | 0.27 |  | 0.13 | 0.61 |
|  |  |  |  |  |  |  |  |
|  | UN | CT/UN25 | 0.11 | 0.31 |  | 0.12 | 0.21 |
|  |  | CT/UN50 | 0.12 | 0.26 |  | 0.10 | 0.35 |
|  |  | UN25/UN50 | 0.13 | 0.53 |  | 0.12 | 0.96 |

**Table S3** The variance inflation factors (VIF) values of the environmental factors in the RDA analysis.

|  | Bacterial communities | | | | |  | Fungal communities | | | | |
| --- | --- | --- | --- | --- | --- | --- | --- | --- | --- | --- | --- |
|  | **Litter** |  | **Organic soil** |  | **Mineral soil** |  | **Litter** |  | **Organic soil** |  | **Mineral soil** |
| Moisture | 1.93 |  | 2.71 |  | 1.83 |  | 1.93 |  | 2.71 |  | 1.83 |
| pH | 2.02 |  | 4.26 |  | 3.25 |  | 2.02 |  | 4.26 |  | 3.25 |
| TC | 1.46 |  | 8.70 |  | 9.85 |  | 1.46 |  | 8.70 |  | 9.85 |
| TN | 3.99 |  | 17.86 |  | 10.09 |  | 3.99 |  | 17.86 |  | 10.09 |
| NH4+ | 3.40 |  | 7.06 |  | 2.68 |  | 3.40 |  | 7.06 |  | 2.68 |
| NO3− | 2.49 |  | 6.79 |  | 5.08 |  | 2.49 |  | 6.79 |  | 5.08 |
| DOC | 10.65 |  | 7.13 |  | 3.54 |  | 10.65 |  | 7.13 |  | 3.54 |
| DON | 13.01 |  | 6.25 |  | 4.18 |  | 13.01 |  | 6.25 |  | 4.18 |
| AP | 3.58 |  | 4.44 |  | 2.83 |  | 3.58 |  | 4.44 |  | 2.83 |

**Fig. S1** Effects of nitrogen addition treatments on the relative abundance of dominant bacterial phyla in different soil layers.


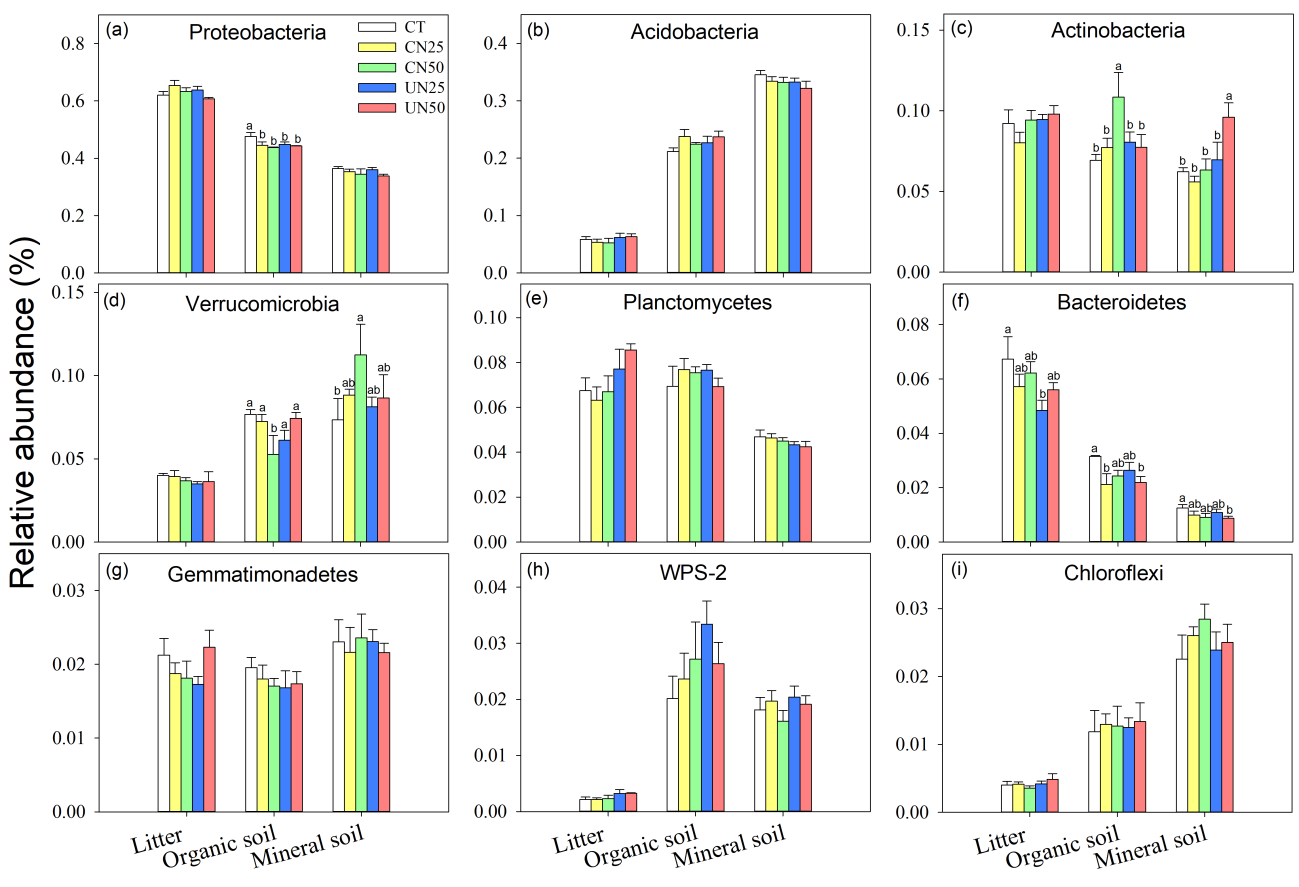


Note: Values are means ± standard error. Within each group of five treatments, values with different letters represent significant differences among the five treatments (*LSD* test, *P* < 0.05). CT, control without N addition; CN25, canopy N addition with 25 kg N ha^−1^ yr^−1^; CN50, canopy N addition with 50 kg N ha^−1^ yr^−1^; UN25, understory N addition with 25 kg N ha^−1^ yr^−1^; UN50, understory N addition with 50 kg N ha^−1^ yr^−1^.

**Fig. S2** Effects of nitrogen addition treatments on the relative abundance of dominant fungal phyla in different soil layers.

**
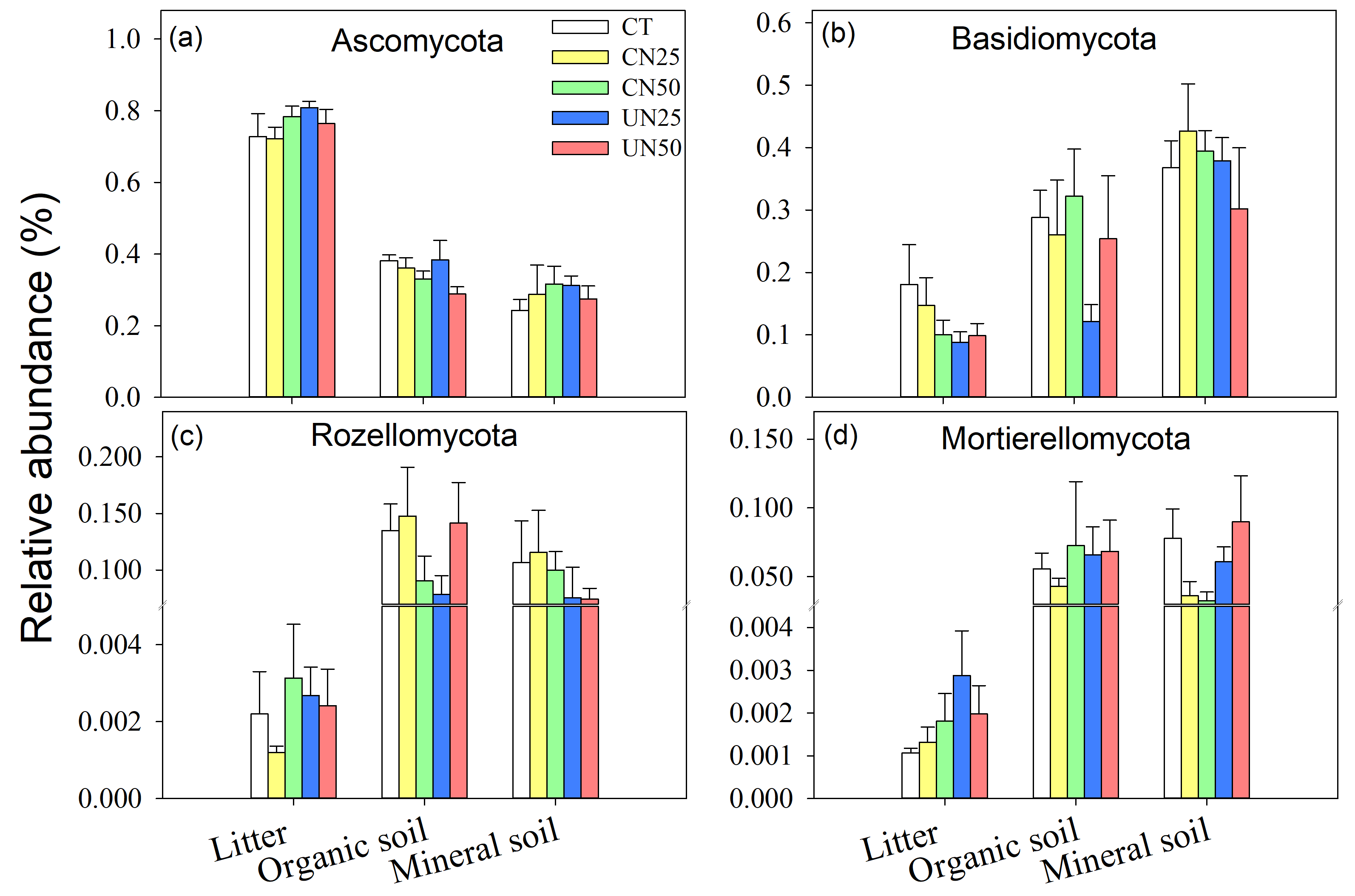
**

Note: Values are means ± standard error. Within each group of five treatments, values with different letters represent significant differences among the five treatments (*LSD* test, *P* < 0.05). CT, control without N addition; CN25, canopy N addition with 25 kg N ha^−1^ yr^−1^; CN50, canopy N addition with 50 kg N ha^−1^ yr^−1^; UN25, understory N addition with 25 kg N ha^−1^ yr^−1^; UN50, understory N addition with 50 kg N ha^−1^ yr^−1^.
